# Making robotic surgery viable and training general surgeons in the Brazilian public healthcare

**DOI:** 10.1007/s11701-026-03645-6

**Published:** 2026-07-29

**Authors:** Gabriela Gouvea Silva, Carlos Dario da Silva Costa, Rodrigo Tavore Strasser, Heitor Zancheta de Andrade, Pedro José Carvalho Tosello, Júlio César André, Jaqueline Venturin, Kátia Luciana Franca Pereira, Samantha Vaccari Grassi Melara, Gedália Pettinelli Tillman, Priscilla Buck de Oliveira Ruiz, Thaina de Oliveira Laluce, Marco Antônio Ribeiro Filho

**Affiliations:** 1 Department of General Surgery, Faculdade de Medicina de São José do Rio Preto (FAMERP), São José do Rio Preto, 15090-000 Brazil; 2 Anesthesiology Department, Faculdade de Medicina de São José do Rio Preto (FAMERP), São José do Rio Preto, Brazil; 3 Center for Studies and Development of Health Education – CEDES, Faculdade de Medicina de São José do Rio Preto (FAMERP), São José do Rio Preto, Brazil; 4 Administration, Hospital de Base FUNFARME/FAMERP, São José do Rio Preto, Brazil; 5 Nursing Department, Faculdade de Medicina de São José do Rio Preto (FAMERP), São José do Rio Preto, Brazil

**Keywords:** Robotic surgery, Healthcare, Hernioplasty, Cholecystectomy

## Abstract

Robotic surgery has become popular in recent decades and is already used in practically all surgical specialties. The increasing use of robotic systems has raised concerns about the safety of patients operated on by surgeons on a learning curve. This demand resulted in a standardized curriculum for training new surgeons. Public hospitals in Brazil possesses robotic systems, making surgical technology available to public healthcare. The relationship between funding and the healthcare model is one of the biggest obstacles to the deployment of expensive new treatments, such as the robotic system. In this prospective study, eighty patients were operated by robotic system, in a Brazilian public hospital, from March 2025 to January 2026, by eight apprentice surgeons. Data concerning demographics, intraoperative information (operation time, and post-operative findings were collected. Patients who underwent surgery were predominantly male (*n* = 48, 60%), with a mean age of 51 years (SD = 15; range: 19–81 years). The procedures performed were 39 inguinal hernioplasties (48.75%), followed by 31 cholecystectomies (38.75%) and other procedures accounted for 11 cases. Complications observed included seroma (*n* = 7), median 30 ml and without any approach required, urinary retention (*n* = 2), one infection of surgical site, one case of prolonged pain after hernioplasty and one conversion to open surgery due to complex inguinoscrotal hernia. Part of the financing came from the apprentice surgeon (70% of total cost), part came from the private initiative (20%), embodied in the robotic enterprise system and finally, funding from public healthcare. The median total cost for 5 inguinal hernioplasties and 5 cholecystectomies was 150000,00 reais, Brazilian currency.

## Introduction

Technological advances in medicine have important impacts on the provision of health services. Robotic surgery has become popular in recent decades and is already used in practically all surgical specialties [1].

Robot-assisted surgery emerged in the 2000s and has grown almost exponentially in the last decade. The use of robotic-assisted surgery has increased 10–40-fold more than that of laparoscopic surgery for general routine procedures [2].

Among the advantages, it offers are the ability to provide virtual information, accuracy in spatial and geometric resolution, greater dexterity, and faster maneuverability. In addition, the ability to operate without fatigue ensures consistent and stable movements, factors that can bring potential advantages, such as reducing blood loss and postoperative recovery time, which may result in better clinical outcomes for patients [3].

The increasing use of robotic systems has raised concerns about the safety of patients operated on by surgeons on a learning curve [4]. This demand resulted in a standardized curriculum for training new surgeons [5].

In Brazil, the regulation of robotic surgery occurred through Resolution 2311/20,226, when the Federal Council of Medicine defined it as “a surgical treatment modality to be used by a minimally invasive route, open or combined, for the treatment of diseases in which its efficacy and safety have already been proven”, establishing the practice as a “highly complex procedure”. The regulation requires that robotic surgeries can only be performed by a physician with a Specialist Qualification Title (SQT) in the surgical area related to the procedure and training in robotic surgery during residency or in specific instruction, One should always remember that robotic surgery is not a new specialty or area of expertise itself, but a new technological tool available to surgeons [3].

The resolution also provides for a main surgeon, responsible for direct patient care regarding diagnosis, choice of technique and intraoperative and postoperative complications, and an instructor-surgeon, responsible for guiding the handling of the robot and evaluating the competence of the main surgeon, not participating directly in patient care. For the main surgeon to be able to perform robotic surgeries without the participation of an instructor-surgeon, they must have completed specific training and performed a minimum of 10 robotic surgeries.

In this defined program, the basic or pre-clinical stage includes acquiring theoretical knowledge about robotic equipment and how the robot works, comprising online training and simulation [6]. In the advanced stage, the apprentice performs the robotic procedure as the main surgeon under the supervision of a surgeon-instructor (proctor) with extensive experience in the technique, to ensure procedures’ safety. After supervising at least ten specialty procedures, the apprentice surgeon’s competence is certified [5].

To ensure proper training for surgeons, one institution shall have the apprentice surgeon, simulation offer, a qualified proctor, a bed-side surgeon, prepared anesthetic and nurse team, complete physical structure of operating rooms containing the robotic platform, and suitable patients.

The first robotic systems acquired in Brazil date back to 2007, covering private institutions. In 2012, public hospitals in Brazil acquired robotic systems, making this surgical technology available in public healthcare [7] based on the principles of universality, integrality, and equality. The relationship between funding and the system’s care model is one of the biggest obstacles to the deployment of expensive new treatments, such as the robotic system [8].

The main limitation for the spread of robotic surgery in Brazil continues to be its cost, evidently higher when compared to laparoscopy [9]. Although there are studies available in other countries concerning costs and viability, Brazilian context is very specific, considering it a middle-income country, an extremely vast territory, estimated population of 213.4 million in 2025 according to IBGE (Brazilian Institute of Geography and Statistics), characterizing a continental country, with public healthcare model very similar to Great Britain’s NHS (National Health Service) and France’s healthcare, in which it was inspired.

Brazil lacks a structured, supervised robotic training program for general surgeons, safe and economically viable, in public healthcare. This article aims to point out the experience and results of a robotic surgical program in a Brazilian public hospital, accounting a hybrid financing.

## Methods

### Study design

In this prospective cohort study (outcome cohort), eighty patients were operated through Da Vinci Xi^®^ robotic system, in Hospital de Base, São José do Rio Preto, São Paulo, Brazil, from March 2025 to January 2026, by eight different apprentice surgeons. All of them had previous certification in General Surgery residency programs. Ethical approval was obtained in Faculty pf Medicine of São José do Rio Preto (FAMERP).

### Participants

Eligibility criteria for patients with cholelithiasis were no previous complications, aptitude to general anesthesia and pneumoperitoneum decided by preoperative assessment. Eligibility criteria for patients with inguinal hernias were the same. Exclusion criteria for inguinal hernioplasty were previous pelvic operations, like prostatectomy. All patients gave written and oral informed consent.

### Economic analysis

Costs were calculated concerning material use, hospital stay, surgical staff payment, medications used. Financing was calculated by adding funding gave by SUS (our public healthcare, *Sistema Único de Saúde*), that covers hospital stay, video laparoscopic material, preoperative assessment and medications; funding from Intuitive Medical^®^, who contributed with subvention in robotic materials; and apprentice surgeon’s funding, covering staff payment and 50% of robotic material cost. Costs with previous structure and basic services like water and electricity did not account for this analysis.

### Training program

Apprentice surgeons followed recommended steps, beginning by online training in specific robotic platform (DaVinci XI^®^), followed by 20 h of simulation and certification process in certified training center after evaluation of trained staff and surgical procedure performed in live model. After these first steps, surgeon had 90 days to perform 10 supervised robotic surgeries.

### Procedures

Data concerning demographics (sex, age, comorbidities), intraoperative information (operation time, anesthesia, hospital stay) and post-operative findings for at least 30 post-operative days (complications such as recurrence for hernia, surgical site infection, urinary retention) were collected. Data was gathered using RedCap^®^ platform and posteriorly submitted to statistical analysis performed in R version 4.1.1 (R Core Team 2021).

### Statistical analysis

Descriptive analyses of the sample were performed, followed by the evaluation of learning curves. Categorical variables were presented as frequencies and percentages. Quantitative variables were described by mean, standard deviation, median, and range.

For the learning curves, the selected measure used was operative time. Each professional’s surgery was ordered chronologically, and a sequential number was assigned (1st, 2nd, 3rd surgery, etc.). The reduction in operative time was calculated by comparing the first and last surgeon’s procedure, in minutes and percentage. The average time in each sequential position was also calculated when data from multiple surgeons were available.

To analyse learning curves, only surgeons with at least 3 recorded cases for each type of procedure were included, since at least three points are needed to characterize a curve.

### Role of the funding source

Partial funding of surgical material from Strattner^®^.

## Results

### Surgeries

The database contained 80 procedures performed by 8 different apprentice surgeons. Initially, the proposal was to perform inguinal hernioplasties, cholecystectomies and eventual procedures that were of surgeons’ interest. As the program unfolded, procedures other than inguinal hernioplasties and cholecystectomies were discontinued, considering these procedures of more complexity, longer operation time and higher learning curves, making them unfit to program’s goals.

Patients who underwent surgery were predominantly male (*n* = 48, 60%), with a mean age of 51 years (SD = 15; range: 19–81 years). The main comorbidity was hypertension (31.25%, *n* = 25), followed by asthma (30%, *n* = 24), diabetes mellitus (15%, *n* = 12), active smoking (11.25%, *n* = 9), and hypothyroidism and dyslipidemia (8.75% each). Other comorbidities included anxiety, depression, prostate benign hyperplasia, and previous neoplasia. The median number of previous surgeries was 1.1, ranging from zero to five previous procedures. These characteristics are shown in Table 1.

**Table 1:** Baseline characteristics

Patients	Data
*Sex*	
Male	48 (60%)
Female	32 (40%)
Age	51 (SD = 15; range: 19–81 years)
*Comorbidities*	
Hypertension	25 (31.25%)
Asthma	24 (30%)
Diabetes Mellitus	12 (15%)
Active smoking	9 (11.25%)
Hypothyroidism	7 (8.75%)
Dyslipidemia	7 (8.75%)
Number of previous abdominal surgery	1.1 (IQS)

Data are n (%), median (IQR), mean (SD), or n/N (%)

The procedures performed were 39 (48.75%) inguinal hernioplasties by TAPP procedure (transabdominal preperitoneal approach), followed by 31 cholecystectomies (38.75%) and other surgeries accounted for 11 cases. These cases were represented by one splenectomy, one Nissen fundoplication, one non-ruled gastrectomy due to GIST (gastrointestinal stromal tumor), one Heller procedure, four eTEPs (enhanced view totally extraperitoneal approach for ventral hernias) and two Ventral TAPPs (transabdominal preperitoneal approach for ventral hernias).

Regarding the type of anesthesia, general anesthesia alone was used in 68.75% of the cases (*n* = 55), while general anesthesia with TAP block was used in 23.75% (*n* = 19). The rate of postoperative complications was 15% (*n* = 12). Complications were classified according to Clavien-Dindo [10] and included accumulation of serous liquid (*n* = 7), median 30 ml and without any approach required, urinary retention (*n* = 2), one superficial infection of surgical site and one conversion to open surgery due to complex inguinoscrotal hernia. The median hospital stay was one day. There was no readmission, reoperation, emergency visits, recurrence for hernia, bile duct injury and 30-day mortality. The longest follow up time was fifteen months.

### Learning curves

**Fig. 1:**
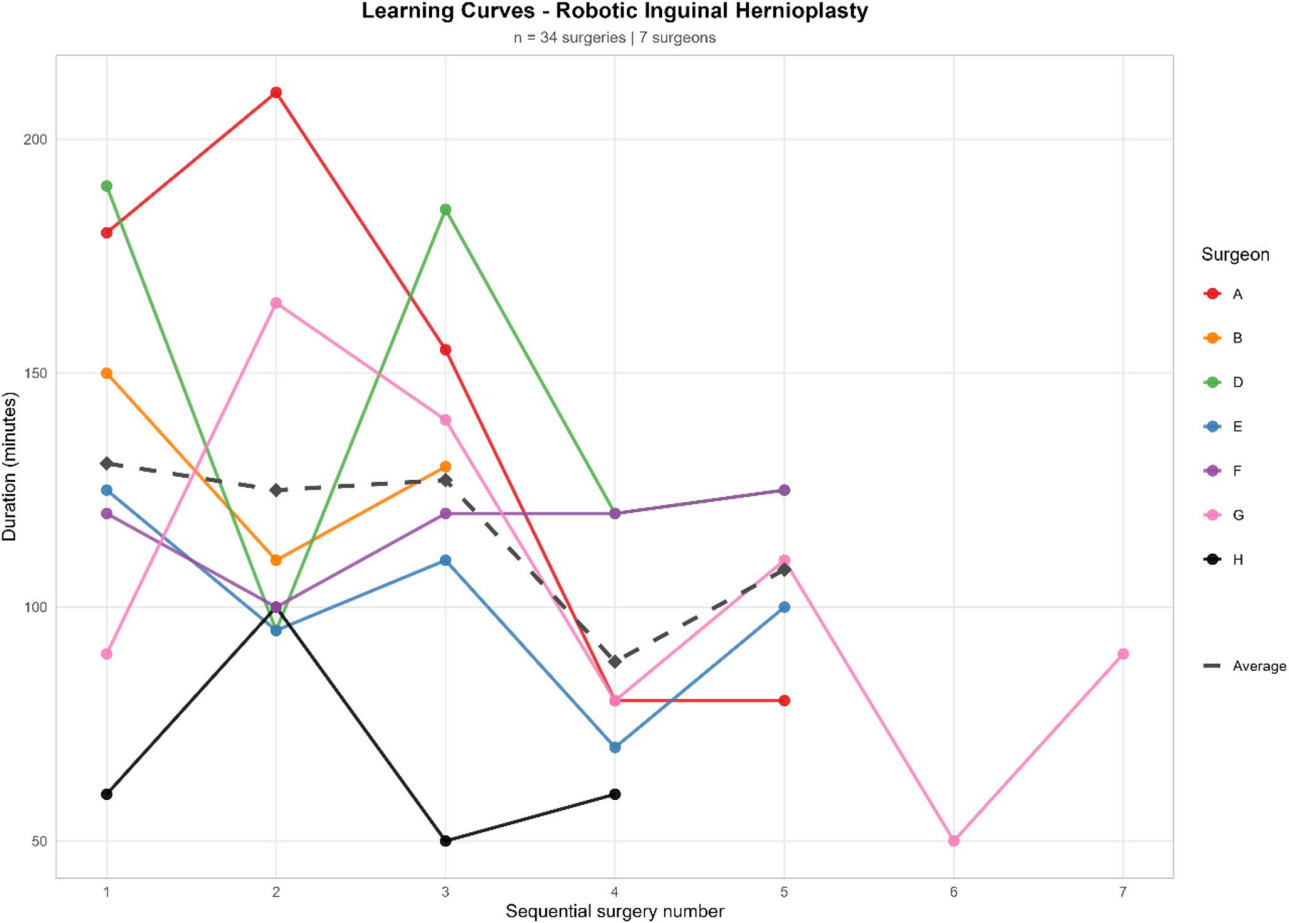
Inguinal hernioplasties performed by eight surgeons, in minutes, in chronological order. The dashed line represents the mean (SD) surgical time among all surgeons in procedures timeline

**Fig. 2:**
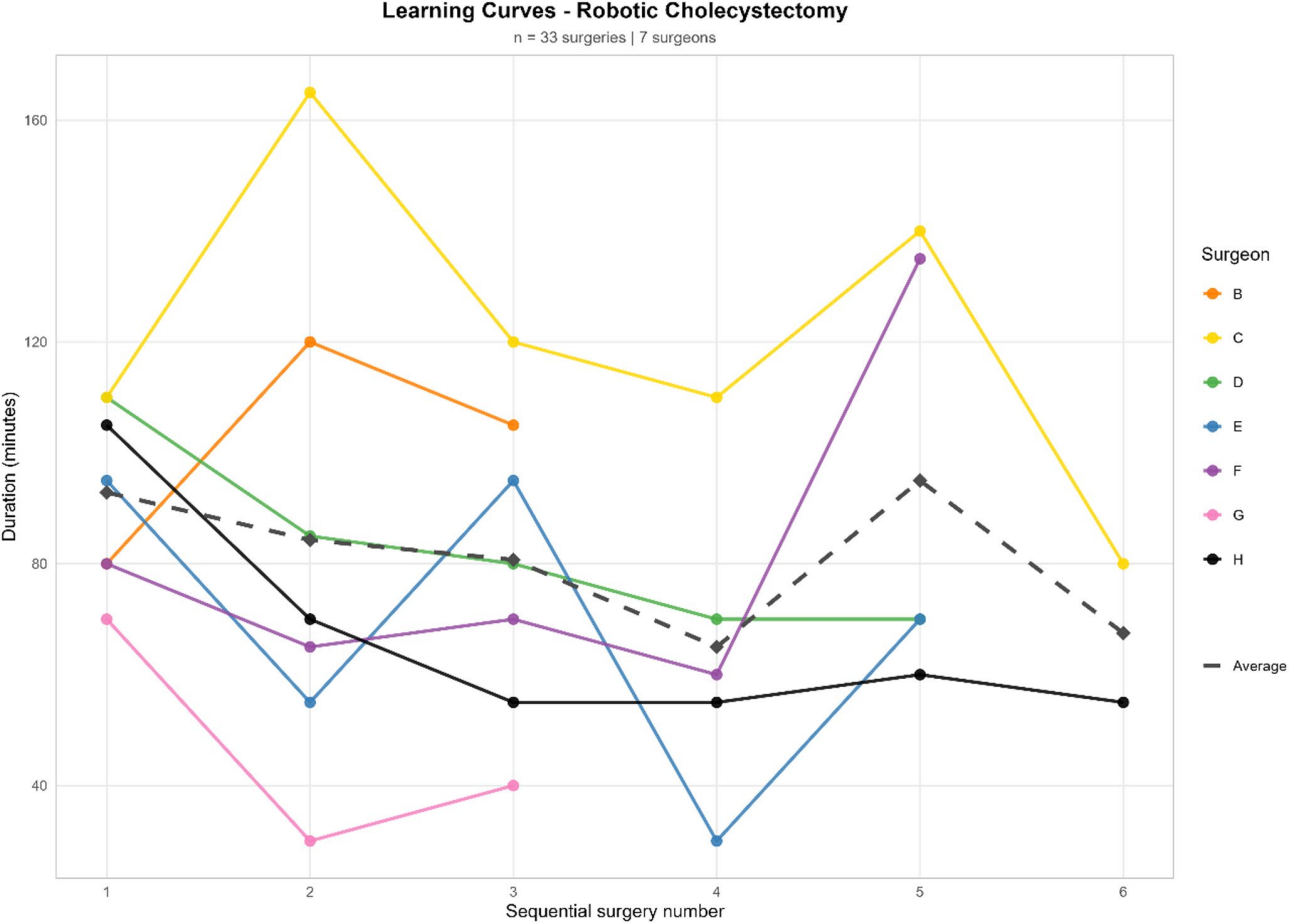
Cholecystectomies performed by eight surgeons, in minutes, in chronological order. The dashed line represents the mean (SD) surgical time among all surgeons in procedures timeline

In learning curves, were considered only inguinal hernioplasties and cholecystectomies. The graphs were generated with ggplot2 version 3.5.1 (Wickham 2016), using fixed colors for each surgeon to facilitate comparison between procedures. Surgeons are represented by lines in different colors and letters. Average time for inguinal hernioplasty decreased from 130 min (min) in the first surgery to 110 min in the fifth procedure. Average time for cholecystectomies decreased from 115 min in the first procedure to 70 min in the fifth procedure. Wide variations in these curves may occur due to specific challenges concerning pathology and patient differences, resulting in technical difficulties (Figs. 1 and 2).

### Costs

**Fig. 3:**
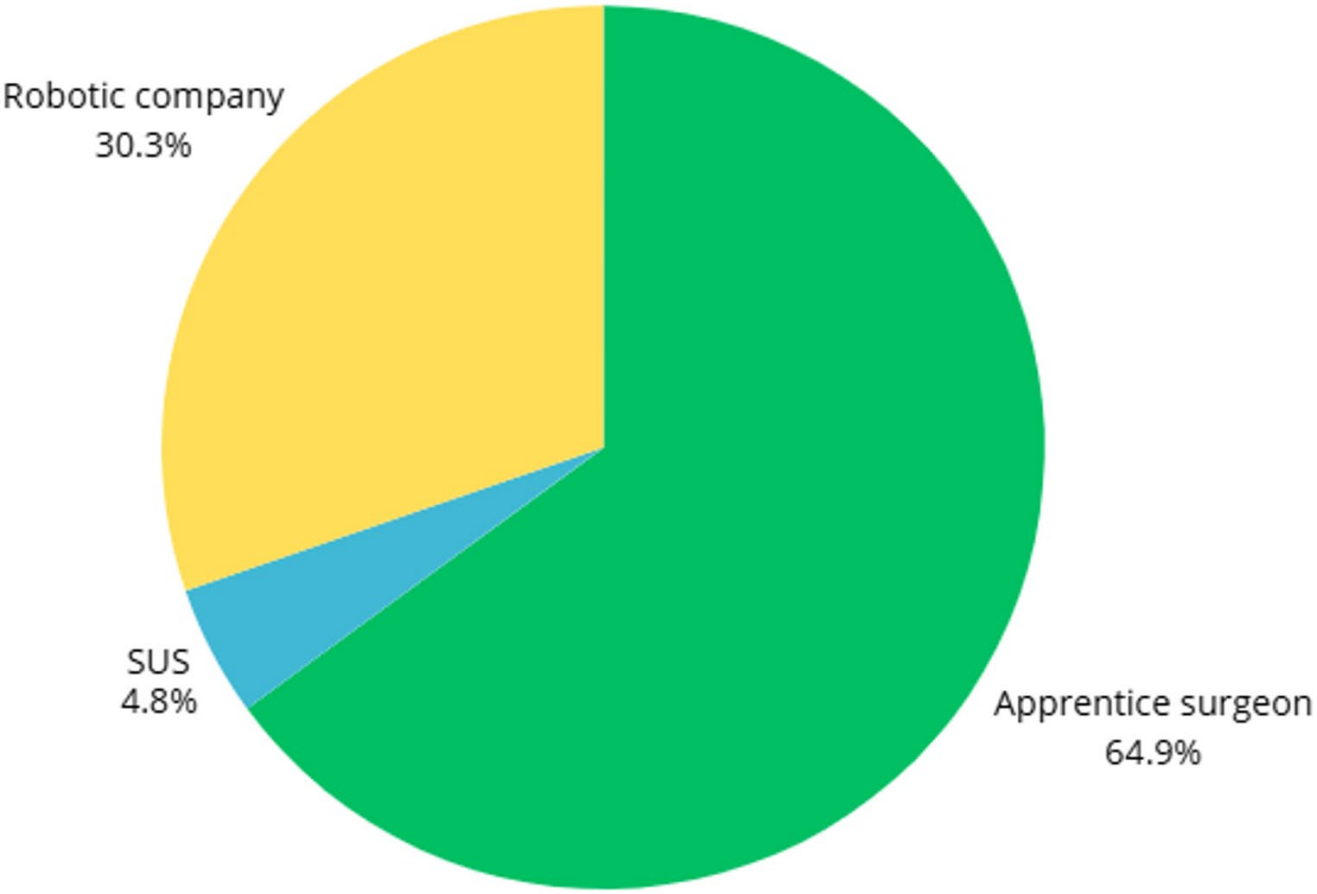
Costs distribution among apprentice surgeon, robotic company and public healthcare (SUS)

The total cost of 5 inguinal hernioplasties and 5 cholecystectomies (cases of surgeons D, E, F) in Brazilian state of São Paulo was R$169.402,10 (Brazilian currency). Part of the financing came from the apprentice surgeon (approximately 64.9% of total cost), a total of R$110.000,00. Part came from the private initiative in form of discount on material purchase by the robotic system distributor (Strattner^®^), R$5750,00 per hernioplasty and R$4500,00 per cholecystectomy, totalizing R$51250,00 in project’s financial support (approximately 30.3%). Funding from public healthcare (SUS) was R$637,97 per hernioplasty and R$992,45 per cholecystectomy, accounting for 4,8% of total and R$8152,10. This data is exhibited in Fig. 3.

## Discussion

According to Cecconelo et al. (2022), if robotic programs do not root in assistance, there will be a significant delay in the spread of trained robotic teams. Proficient surgeons and nurses in robotic surgery are the main modifying factors of the cost equation in robotics [11].

After identifying sources of expenditure that were blocking the development of a robotic program in our public institution as a middle-income country, a model combining public and private funding was schematized and put into practice an alternative to solve the cost issue. The most common manner to become a robotic surgeon in Brazil, is to finance the surgeon alone the settled program of the Brazilian Federal Council of Medicine, reducing significantly the access of surgeons to this technology.

The proposal here described was economically viable only for simpler procedures, as inguinal hernioplasties and cholecystectomies, disregarding the larger procedures made, where there was no economic feasibility when considering procedures costs alone. There are, although, some studies that point out that despite the high costs of robotic surgery, the decrease of hospital stay, pain and lower complications make it viable [12].

The apprentice surgeons usually request to join the program as an upgrade in their surgical career, to be updated in technology and skills, aiming to their patients offer better care. Since in Brazil there is not currently a program that encompasses robotic training and health education, neither in residency programs nor continuing education, the financial model proposed may be an alternative to democratize robotic surgery [13].

The complications’ type and incidence match the current acceptable literature [14–16], ensuring safety to the procedures, even considering learning curves. Proficient robotic surgeons tend to operate inguinal hernias for 45 to 60 min [17], and cholecystectomies between 68 and 82 min [18], matching findings in this study, that despite the small number of operated cases per surgeon, may be explained by the presence and assistance of both experienced proctor and bed-side surgeons.

It is possible to point out the early feasibility of the proposal, bringing benefits to patients, capacitation and stimulation of cohesion within the surgical team, and promoting training to surgeons not only of the same institution but for surgeons from different parts of the country.

We believe that this is proof that implementing robotic surgery programs in public hospitals is possible and may accelerate surgical modernization and better surgical care in our country.

Limitations of this study are mainly related to the selected procedures for the program. Patients were carefully selected, complex cases were discontinued, procedures were simple, and proctors/bedside surgeons were present. These factors likely explain the acceptable morbidity and operative times, but they also limit external validity. This bias probably diminished surgical time and possibly explains our low complications rates. Other limitations include single center study, sample size, and reduced post-operative following. Furthermore, although effective, this funding model raises questions about equity in access to training for surgeons with limited resources, an important consideration for future expansion. This aspect may also point out a possible selection bias of apprentice surgeons, whose financial capability is key, beside prior skills and elevated motivation, highlighting the need for further studies in profiling these candidates and improving access.

The presented model may impact on future applications as it can be expanded to other hospitals and may comprehend more complex procedures as surgeons surpass their initial training, according to every institution structure and necessity over time.

## Data Availability

No datasets were generated or analysed during the current study.

## Declarations

### Copyright

Protected material, with signed permission statements from author and publisher.

## References

[1] Leal Ghezzi T, Campos Corleta O (2016) 30 Years of Robotic Surgery. World J Surg 40(10):2550–2557. 10.1007/s00268-016-3543-9. PubMed PMID: 27177648 27177648 10.1007/s00268-016-3543-9

[2] Armijo PR, Pagkratis S, Boilesen E, Tanner T, Oleynikov D (2018) Growth in robotic-assisted procedures is from conversion of laparoscopic procedures and not from open surgeons’ conversion: a study of trends and costs. Surg Endosc 32(4):2106–2113. 10.1007/s00464-017-5908-z 29067582 10.1007/s00464-017-5908-z

[3] Trindade EN, Teixeira ELF, Bortolin VS, Difante LDS, Trindade MRM (2024) Considerações éticas e legais do uso da cirurgia robótica no Brasil. Rev Col Bras Cir 51. 10.1590/0100-6991e-20243787

[4] Shah D, Tesfai FM, Boal M, Arezzo A, Francis N (2025) Evaluation of current and emerging endoluminal robotic platforms using the IDEAL framework. Minimally Invasive Therapy and Allied Technologies. Taylor and Francis Ltd., pp 253–266. PubMed PMID: 39985163 10.1080/13645706.2025.2467805 39985163

[5] de Araujo PHXN, Pêgo-Fernandes PM (2023) Robotic surgery training. Sao Paulo Medical Journal. Associacao Paulista de Med. 10.1590/1516-3180.2022.1415310823. PubMed PMID: 37909546 10.1590/1516-3180.2022.1415310823 PMC10619944 37909546

[6] Klok JW, Rahimi M, Hardon S, Postema R, Bonjer J, Daams F et al (2025) The impact of simulated intra-abdominal movement on basic laparoscopic skills development: a feasibility study. Minim Invasive Ther Allied Technol 34(4):324–333 PubMed PMID: 40119638 40119638 10.1080/13645706.2025.2481394

[7] Morrell ALG, Morrell-Junior AC, Morrell AG, Mendes JMF, Tustumi F, De-Oliveira-e-silva LG et al (2021) The history of robotic surgery and its evolution: When illusion becomes reality. Rev Col Bras Cir 48:1–9. 10.1590/0100-6991e-20202798. PubMed PMID: 33470371 10.1590/0100-6991e-20202798 PMC10683436 33470371

[8] Costa TN, Tustumi F, Maia Ferros LS, Colonno BB, Abdalla RZ, Ribeiro-Junior U et al (2022) Robotic-assisted versus laparoscopic incisional hernia repair: differences in direct costs from a brazilian public institute perspective. Arquivos Brasileiros de Cirurgia Digestiva 35. 10.1590/0102-672020220002e1714. PubMed PMID: 36629691 10.1590/0102-672020220002e1714 PMC9831626 36629691

[9] Araujo RLC, Benevenuto DSá., Zilberstein B, Sallum RA, Aguiar S, Cavazzola LT et al (2020) Overview and perspectives about the robotic surgical certification process in Brazil: The new statement and a national web-survey. Rev Col Bras Cir 47:1–8. 10.1590/0100-6991E-20202714. PubMed PMID: 33111834 10.1590/0100-6991e-20202714 33111834

[10] Katayama H, Kurokawa Y, Nakamura K, Ito H, Kanemitsu Y, Masuda N et al (2016) Extended Clavien-Dindo classification of surgical complications: Japan Clinical Oncology Group postoperative complications criteria. Surg Today. ;46(6):668–85. 10.1007/s00595-015-1236-x. PubMed PMID: 26289837 10.1007/s00595-015-1236-x PMC4848327 26289837

[11] Nayeemuddin M, Daley SC, Ellsworth P (2013) Modifiable factors to decrease the cost of robotic-assisted procedures. AORN J 98(4):343–352 012. PubMed PMID: 24075331 24075331 10.1016/j.aorn.2013.08.012

[12] Tang Y, Dou B (2025) Cost-effectiveness analysis of robotic surgery in healthcare for older individuals: a systematic review based on randomized controlled trials. Front Public Health Front Media SA. 10.3389/fpubh.2025.1614654. PubMed PMID: 40880918 10.3389/fpubh.2025.1614654 PMC12381784 40880918

[13] Ishibayashi K, Saito H, Fujimori D, Saito H, Yamaguchi T, Ohbatake Y et al (2026) Evaluation of the learning effect for ArtiSential^®^ articulating laparoscopic instrument: back to the manual laparoscopic forceps. Minim Invasive Therapy Allied Technol 35(1):1–7. 10.1080/13645706.2025.2582210 10.1080/13645706.2025.2582210 41385217

[14] Friis-Andersen H, Bisgaard T (2016) The Danish inguinal hernia database. Clinical Epidemiology. Dove Medical Press Ltd, pp 521–524. doi:10.2147/CLEP.S99512 10.2147/CLEP.S99512 PMC5096723 27822094

[15] Simons MP, Smietanski M, Bonjer HJ, Bittner R, Miserez M, Aufenacker TJ et al (2018) International guidelines for groin hernia management. Hernia. ;22(1):1–165. 10.1007/s10029-017-1668-x. PubMed PMID: 29330835 10.1007/s10029-017-1668-x PMC5809582 29330835

[16] Simons MP, Aufenacker T, Bay-Nielsen M, Bouillot JL, Campanelli G, Conze J et al (2009) European Hernia Society guidelines on the treatment of inguinal hernia in adult patients. Hernia 343–403. 10.1007/s10029-009-0529-7. PubMed PMID: 19636493 10.1007/s10029-009-0529-7 PMC2719730 19636493

[17] Solaini L, Cavaliere D, Rocco G, Avanzolini A, Di Pietrantonio D, Ercolani G (2023) Differences in the learning curve of robotic transabdominal preperitoneal inguinal hernia repair according to surgeon’s robotic experience. Hernia 27(5):1123–1129. 10.1007/s10029-023-02846-4. PubMed PMID: 37592165 37592165 10.1007/s10029-023-02846-4 PMC10533585

[18] Vidovszky TJ, Smith W, Ghosh J, Ali MR (2006) Robotic Cholecystectomy: Learning Curve, Advantages, and Limitations. J Surg Res 136(2):172–178. 10.1016/j.jss.2006.03.021 17059837 10.1016/j.jss.2006.03.021

